# Impact of Nanomaterials in Biological Systems and Applications in Nanomedicine Field

**DOI:** 10.3390/nano12101775

**Published:** 2022-05-23

**Authors:** Valeria De Matteis, Mariafrancesca Cascione, Stefano Leporatti

**Affiliations:** 1 Department of Mathematics and Physics “Ennio De Giorgi”, University of Salento, Via Arnesano, 73100 Lecce, Italy; mariafrancesca.cascione@unisalento.it; 2 CNR Nanotec—Institute of Nanotechnology, Via Monteroni, 73100 Lecce, Italy; stefano.leporatti@nanotec.cnr.it

The increasingly widespread use of engineered nanomaterials in many applications increases the need to understand the mechanisms behind their toxicity. The aim of this Special Issue is to investigate the onset of different pathways following the interactions between nanoparticles (NPs) and biological systems such as cells, microorganisms, and animal models. The physicochemical properties of inorganic and organic NPs strongly affect their toxic behavior causing alterations in cell biochemical pathways, cytoskeleton reorganization, as well as different biodistribution in organs and tissues [1,2,3]. The study of nanotoxicity is critical for the customization of specific therapies based on nano-systems against certain diseases and microorganisms [4,5,6,7]. Tripathi et al. [8], reported an easy and eco-friendly chemical route to obtain triangular and hexagonal anionic gold NPs (AuNPs), with dimensions in the range of (61–178) nm, in order to understand their toxicity on 3T3-L1 adipocytes. The authors demonstrated that the triangular shape was internalized more efficiently than spherical NPs, showing a shape-dependent uptake. The negative zeta potential offered the possibility of functionalizing the AuNPs using positive-charged molecules for drug delivery applications. In addition, the cell viability assessment confirmed the negligible toxicity of these materials in cells. An eco-friendly route was used to obtain Carbon Nanodots (CNDs), assessing their anti-inflammatory effects on Human Microvascular Endothelial Cells (HMEC-1) by Belperain et al. [9]. Using non-cytotoxic concentrations (0.001, 0.03, 0.1, 0.3, 0.6, and 1.2 mg/mL), a reduction in pro-inflammatory cytokines, such as Interleukin-8 (IL-8) and Interleukin 1 beta (IL-1β) was demonstrated.

Specific types of NPs can be applied as a filler to improve specific properties of materials that are commonly used in dental and mandibular prosthetic implants, such as poly(methyl-methacrylate) (PMMA). Cascione et al. [10] added Titanium Dioxide NPs (TiO_2_NPs) and Halloysite Clay Nanotubes (HNTs) in PMMA matrix. This addition reduced the PMMA roughness and increased the wettability: these two conditions are involved in the decrease in *Candida albicans* colonization, the most common yeast-inducing infections in oral cavities. El-Saadony et al. [11], developed an innovative solution to contrast agricultural pests caused by *Tribolium castaneum*, which provokes crop reductions and contamination entering in human and animal food chains. The authors achieved copper NPs (CuNPs) by *Pseudomonas fluorescens* MAL2 with a size ranging from 10 to 70 nm, demonstrating the efficacy of these NPs against *Tribolium castaneum* at low doses with an LC_50_ equal to 693.7 ppm after 24 h of exposure. Zaho et al. [12] developed a vaccine delivery platform consisting of polymer-coated liposomes and polyelectrolyte complexes (PECs) to transport lipopeptide subunit vaccine (LCP-1) against group *A Streptococcus* in outbred Swiss mice. They concluded that the PEC system, especially when containing heparin and alginate/trimethyl chitosan (TMC), could be a powerful candidate for intranasal administration of peptide-based subunit vaccine delivery. Staying on the topic of microorganisms, Saleem and colleagues [13] have drawn up a robust review of the nanotechnology-based techniques and products against *Leishmaniasis*, a protozoan vector-born disease affecting millions of people worldwide. They collected the latest drug delivery strategies based on liposomes, lipid nanocapsules, metal and metallic oxide NPs, polymeric NPs, nanotubes and nanovaccines. The guest editors of this Special Issue would like to acknowledge all the authors for their scientific support and willingness to share their knowledge from different fields of investigation. Moreover, the editors would like to express their appreciation to the peer reviewers for their accurate analysis of manuscripts—effectively contributing to the publication of the Special Issue—and the managing editors of *Nanomaterials,* for their continuous support.
